# Effects of *Helicobacter pylori* Infection on the Oral Microbiota of Reflux Esophagitis Patients

**DOI:** 10.3389/fcimb.2021.732613

**Published:** 2021-09-16

**Authors:** Tian Liang, Fang Liu, Lijun Liu, Zhiying Zhang, Wenxue Dong, Su Bai, Lifeng Ma, Longli Kang

**Affiliations:** ^1^Key Laboratory for Molecular Genetic Mechanisms and Intervention Research on High Altitude Disease of Tibet Autonomous Region, School of Medicine, Xizang Minzu University, Xianyang, China; ^2^Key Laboratory of High Altitude Environment and Genes Related to Diseases of Tibet Autonomous Region, School of Medicine, Xizang Minzu University, Xianyang, China

**Keywords:** Reflux esophagitis, oral microbiota, *Helicobacter pylori*_3_, alpha diversity, beta diversity

## Abstract

The human oral microbiota plays a vital role in maintaining metabolic homeostasis. To explore the relationship between *Helicobacter pylori* (*Hp*) and reflux esophagitis, we collected 86 saliva samples from reflux esophagitis patients (RE group) and 106 saliva samples from healthy people (C group) for a high-throughput sequencing comparison. No difference in alpha diversity was detected between the RE and the C groups, but beta diversity of the RE group was higher than the C group. Bacteroidetes was more abundant in the RE group, whereas Firmicutes was more abundant in the C group. The linear discriminant analysis effect size analysis demonstrated that the biomarkers of the RE group were *Prevotella*, *Veillonella*, *Leptotrichia*, and *Actinomyces*, and the biomarkers of the C group were *Lautropia*, *Gemella*, *Rothia*, and *Streptococcus*. The oral microbial network structure of the C group was more complex than that of the RE group. Second, to explore the effect of *Hp* on the oral microbiota of RE patients, we performed the ^14^C-urea breath test on 45 of the 86 RE patients. We compared the oral microbiota of 33 *Hp*-infected reflux esophagitis patients (REHpp group) and 12 non-Hp-infected reflux esophagitis patients (REHpn group). No difference in alpha diversity was observed between the REHpn and REHpp groups, and beta diversity of the REHpp group was significantly lower than that of the REHpn group. The biomarkers in the REHpp group were *Veillonella*, *Haemophilus*, *Selenomonas*, *Megasphaera*, *Oribacterium*, *Butyrivibrio*, and *Campylobacter*; and the biomarker in the REHpn group was *Stomatobaculum*. *Megasphaera* was positively correlated with *Veillonella* in the microbial network of the REHpp group. The main finding of this study is that RE disturbs the human oral microbiota, such as increased beta diversity. *Hp* infection may inhibit this disorderly trend.

## Introduction

The oral microbiota is closely related to the esophageal microbiota, and the esophageal microbiota is largely affected by the oral microbiota (Lv et al., 2019). Reflux esophagitis (RE) can cause disorder of the host oral microbiota (Wang et al., 2020). Disturbances in the oral microbiota are influential in inducing diseases of the upper and lower gastrointestinal tract (Schmidt et al., 2019). The esophagus and oral mucosa are damaged during acid reflux, and the oral microbiota of RE patients differs from that of healthy people (Wang et al., 2020). In addition, oral bacteria may migrate to the esophagus, thereby indirectly affecting the esophageal microorganisms and promoting the occurrence and development of esophageal diseases (Gao et al., 2018). Human oral microbiota disorders can induce chronic inflammation through immune pathways, leading to the occurrence of RE, Barrett’s esophagus, and esophageal adenocarcinoma (Snider et al., 2016).

*Helicobacter pylori* (*Hp*) is one of the most important causes of chronic gastritis, gastric atrophy, and gastric cancer (Ding, 2020). Previous studies have reported that *Hp* infection can induce disorders of the oral and stomach microbiota in humans (Zhao et al., 2019). However, *Hp* infection has a protective effect on patients with esophageal diseases (Sugimoto et al., 2020). The oral and esophageal mucosa and microbiota of RE patients are damaged by acid reflux. Gastric atrophy occurs when the body is infected with *Hp*, which, in turn, decreases gastric acid, thereby indirectly protecting the oral mucosa (Ding, 2020). The incidence of esophageal disease may increase after eradicating *Hp*. Serum ghrelin levels increase during *Hp* eradication, affecting gastric emptying, and ultimately leading to an increased risk of esophageal reflux disease. Other studies have found that *Hp* infection is negatively correlated with the incidence of esophageal diseases, such as Barrett’s esophagus and esophageal adenocarcinoma (Anderson et al., 2008).

Only a few studies have assessed the effect of *Hp* infection on the oral microbiota of patients with RE, so we carried out this study. The purpose of this study is to reveal the effect of *Hp* infection on the oral microbiota of patients with RE and to lay the foundation for investigating the interaction mechanism between *Hp* and RE.

## Materials and Methods

### Sample Collection

We recruited 86 RE patients and 106 healthy volunteers at Xizang Minzu University Affiliated Hospital from May 2018 to July 2019. All participants were diagnosed by endoscopy using the Los Angeles classification method. All RE patients had typical symptoms of heartburn and reflux. The inclusion criteria were no treatment with glucocorticoids, antibiotics, proton pump inhibitors, or other drugs within 3 months; no gastrointestinal diseases or cancer; no gastrointestinal surgery; no oral diseases, and no pregnancy. The ^14^C-urea breath test was used to identify whether patients with RE were infected with *Hp*. We collected 10 mL of saliva from volunteers in a 50 mL sterile tube and stored it at −80°C until further use. We also collected information on gender, age, height, weight, body mass index (BMI), and eating habits. All participants signed informed consent and the purpose of the study was understood by all. This study was reviewed and approved by the Ethics Committee of Xizang Minzu University before the participants were recruited (ID: 201702). The experiments followed the standard biosecurity and safety procedures of Xizang Minzu University.

### DNA Extraction and High-Throughput Sequencing

The TIANamp Swab DNA Kit (Shanghai, China) was used to extract total microbial DNA. The V3-V4 region of the 16S rRNA gene was amplified with universal 341F and 805R primers. Polymerase chain reaction amplification, purification, and quantification were performed as described previously (Liu F. et al., 2021). The DNA library was merged into equimolar concentrations and sequenced using an Illumina MiSeq platform with a 2 × 250 paired-end protocol. The raw sequence data were deposited at the Genome Sequence Archive at the Data Center, Beijing Institute of Genomics, Chinese Academy of Sciences, under Accession number CRA: CRA004395, which is publicly accessible at https://ngdc.cncb.ac.cn/gsa.

### Bioinformatics and Statistical Analysis

Raw FASTQ files were demultiplexed, quality-filtered using Trimmomatic v0.39 (Bolger et al., 2014), and merged with FLASH v1.2.11 (Magoc and Salzberg, 2011) as described previously (Liu F. et al., 2021). Operational taxonomic units (OTUs) were defined at 97% similarity by UPARSE v7.1 (Edgar, 2013), and chimeric sequences were detected and removed with UCHIME v4.1 (Edgar et al., 2011). Taxonomy was assigned by the RDP Classifier algorithm v2.2 against the Silva (SSU123) 16S rRNA database using a confidence threshold of 80%. Species accumulation curves were constructed with the ‘vegan’ package (Oksanen et al., 2013). Alpha diversity was calculated using the Chao 1 and Shannon indices (Schloss et al., 2009). Principal coordinate analysis (PCoA) and permutational multivariate analysis of variance were performed to determine beta diversity (Bray-Curtis distance) (Lozupone and Knight, 2005; Kelly et al., 2015). A linear discriminant effect size analysis (LEfSe software v1.0, Segata et al., 2011) was used to discover biomarkers between different groups (LDA score > 2.0). The demographic information of the different groups was compared with Student’s *t*-test. The chi-square test was used to verify the difference in eating habits between the groups. The relative abundance of bacterial phyla and genera between the different groups was compared using the Wilcoxon rank-sum test. The false discovery rate (FDR) was used to correct the *P*-values, and *P*-FDR < 0.05 was considered significant. SparCC and 100 bootstraps were used to calculate the correlation of each oral bacteria genus in the co-occurrence network between different groups (correlation value > 0.4, *P* value < 0.05). Centrality of the co-occurrence network was calculated using closeness and eigenvector of the node (Friedman and Alm, 2012; Wang et al., 2018). Gephi software was used to visualize the oral microbiota co-occurrence networks (Wang et al., 2018).

## Results

A total of 192 participants were recruited between May 2018 and July 2019, including 86 RE patients and 106 healthy subjects, and a total of 192 saliva samples were collected. No significant differences in height, weight, or BMI were observed between the RE and C groups (*P* > 0.05). The dietary habits of the groups were similar, but the RE group consumed less fiber and had a higher smoking rate (*P* < 0.05), as shown in **Table 1**.

**Table 1:** Basic information of Reflux Esophagitis (RE) patients and healthy controls.

Characteristics	Healthy controls	RE patients	*χ* ^2^	*P* value
Gender				
Male	49	50	1.09	*P > 0.05*
Female	57	36	1.09	*P > 0.05*
Height	165.38 ± 7.38	164.9 ± 7.13		*P > 0.05*
Weighten	63.96 ± 10.2	63.76 ± 10.83		*P > 0.05*
Body mass index (BMI)	23.3 ± 2.66	23.35 ± 2.9		*P > 0.05*
Age	50.81 ± 13.9	56.27 ± 13.6		*P > 0.05*
Smoking				
None or occasionally	90	50	7.79	*P < 0.05*
sometimes or frequently	16	36	7.79	*P < 0.05*
Alcohol drinking				
None or occasionally	85	58	1.5	*P > 0.05*
sometimes or frequently	21	28	1.5	*P > 0.05*
Fiber intake				
Low fiber intake	47	64	7.05	*P < 0.05*
High fiber intake	59	22	7.05	*P < 0.05*
Salt intake				
Low salt intake	104	81	1.76	*P > 0.05*
High salt intake	2	5	1.76	*P > 0.05*
Fat and meat intake				
Low fat intake	94	70	1.1	*P > 0.05*
High fat intake	12	16	1.1	*P > 0.05*

### Comparison of Community Composition of the Oral Microbiota Between the RE and C Groups

**Figure 1:**
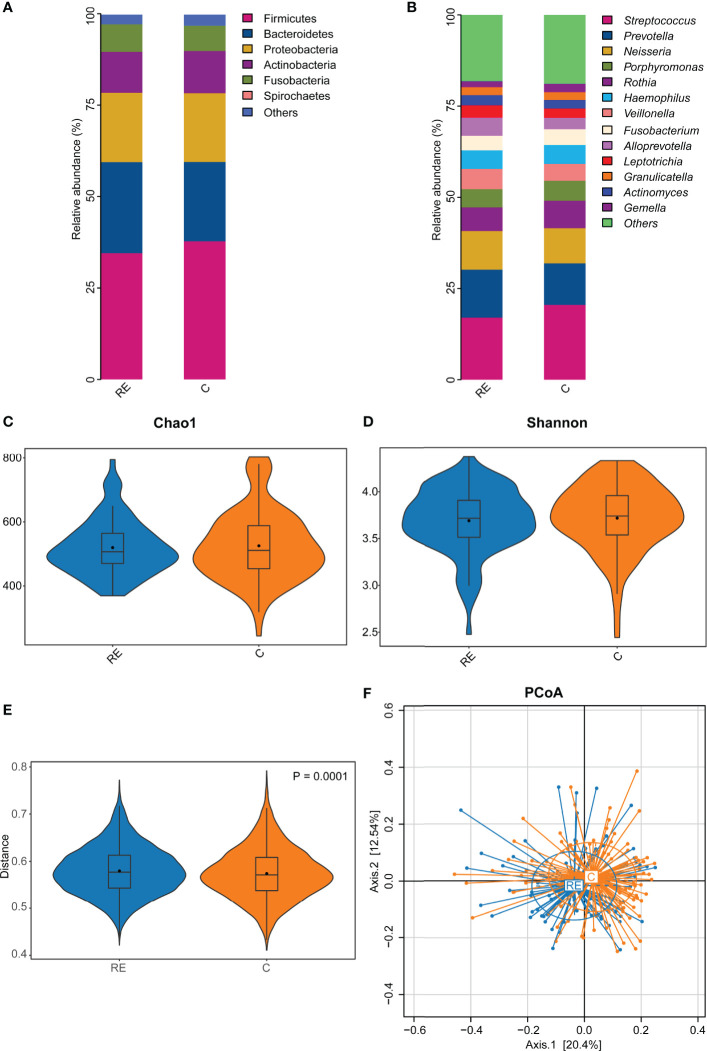
The effect of reflux esophagitis on the human oral microbiota. The oral bacterial community composition in reflux esophagitis patients (RE group) and healthy people (C group) at the phylum level **(A)**, and the genus level **(B)**. The alpha diversity of the RE group tended to be lower than that of the C group, but it was not significantly different, *P* > 0.05 **(C, D)**. The beta diversity of RE group was significantly higher than that of the C group, R^2^ = 0.009, *P* = 0.0001 **(E, F)**.

A total of 4,785,985 sequences were obtained after quality control, and the average reads per sample were 71,432 (range 43,299-98,661 reads per sample), and 71,261 OTUs were obtained. The rarefaction curve showed that sequencing depth captured most of the bacterial species in the oral microbiota sample (**Supplementary Figure 1**). The human oral microbiota was dominated by Firmicutes (36.01%), Bacteroidetes (23.66%), Proteobacteria (19.80%), Actinobacteria (9.59%), Fusobacteria (7.40%), and Spirochaetes (1.31%) (**Figure 1A**).

At the genus level, the oral microbiota was composed primarily of *Streptococcus* (19.15%), *Prevotella* (11.71%), *Neisseria* (10.16%), *Porphyromonas* (6.15%), *Rothia* (6.12%), *Haemophilus* (5.48%), *Veillonella* (4.60%), *Fusobacterium* (4.49%), *Alloprevotella* (4.05%), *Leptotrichia* (2.78%), *Granulicatella* (2.18%), *Actinomyces* (2.08%), and *Gemella* (2.03%) (**Figure 1B**).

### Diversity of the Oral Microbiota in the RE and C Groups

All 86 RE patients were compared to the 106 controls to test the potential effect of RE on the oral microbiota. No significant difference in alpha diversity was observed between the RE and C groups (*P* = 0.61, **Figures 1C, D**). However, the beta diversity of the C group was significantly lower than that of the RE group (*P* = 0.0015, R^2^ = 0.041, **Figure 1E**). The PCoA plots revealed significantly different community structures between the RE and C groups (**Figure 1F**).

### Comparison of Oral Bacterial Abundance Between the RE and C Groups

**Figure 2:**
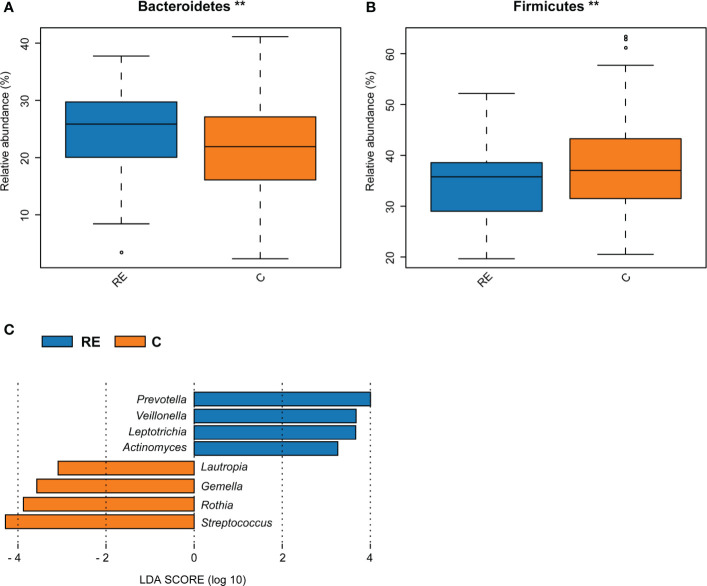
Comparison of bacterial abundance between the reflux esophagitis and the healthy groups. The abundance of Bacteroidetes in the RE group was higher, while the abundance of Firmicutes in the C group was higher, **P < 0.01 **(A, B)**. LEfSe analysis results of the RE and C groups **(C)**.

Bacteroidetes were more abundant in the RE group, whereas Firmicutes were more abundant in the C group (**Figures 2A, B**). **Supplementary Figure 2A** indicates no significant differences at the genus level between the RE and C groups. The LEfSe analysis screened out biomarkers with significant abundance between the RE and C groups. The abundance rates of *Prevotella*, *Veillonella*, *Leptotrichia*, and *Actinomyces* were higher in the RE group; and those of *Lautropia*, *Gemella*, *Rothia*, and *Streptococcus* were higher in the C group (LDA > 2, *P* < 0.05, **Figure 2C**).

### Comparison of the Oral Microbial Network Between the RE and C Groups

**Figure 3:**
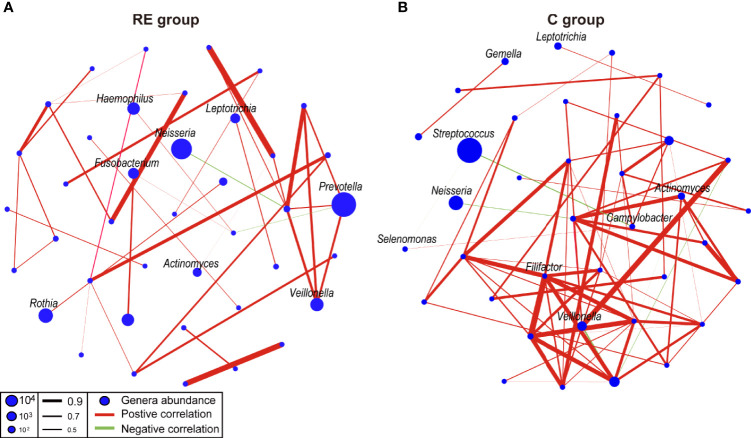
Co-occurrence networks of the reflux esophagitis and healthy groups **(A, B)**. The co-occurrence network was inferred by the pairwise correlation of the relative abundances of all genera. Each node in the network represents a bacterial genus. The node size represents the mean relative abundance of a genus in the oral microbiota. Red line: positive correlation; green line: negative correlation. Relative thickness of the lines represents the degree of the correlation (greater thickness of the edges means a stronger correlation).

We compared the oral microbial network of the RE and C groups to explore the effect of RE on the oral microbial network (**Figure 3**). As results, the microbial network of the RE group (40 nodes, 38 edges) had more edges than the microbial network of the C group (38 nodes, 71 edges). *Prevotella* was positively correlated with *Veillonella*, while *Neisseria* was negatively correlated with *Atopobium* in the RE group. *Streptococcus* was negatively correlated with *Selenomonas* and *Campylobacter* in the C group (**Figure 3**). These results suggest that the microbial network of the human oral microbiota was affected by reflux esophagitis.

### Diversity of the Oral Microbiota in the REHpp and REHpn Groups

**Figure 4:**
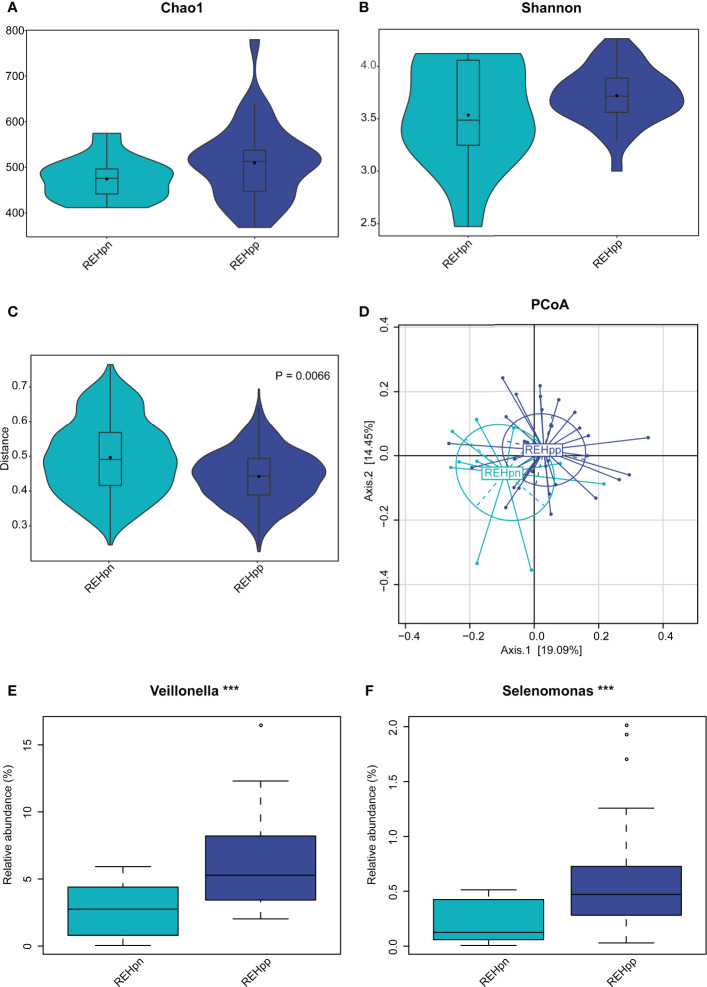
The influence of *Hp* infection on the oral microbiota of reflux esophagitis patients. The alpha diversity of the REHpp group tended to be higher than that of the REHpn group, but it was not significant, *P* > 0.05 **(A, B)**, and the beta diversity was significantly lower than that of the REHpn group, R^2^ = 0.053, *P* = 0.0066 **(C, D)**. Comparison of bacterial abundance between the REHpn and REHpp groups at the genus level **(E, F)**, the abundances of *Veillonella* and *Selenomonas* in the REHpp group were significantly higher than those in the REHpn group, ***P < 0.005.

We further explored the effect of *Hp* infection on the oral microbiota of RE patients and compared 33 RE patients with *Hp* infection (REHpp) and 12 RE patients without *Hp* infection (REHpn). As a result, no significant difference in alpha diversity was observed between the REHpn and REHpp groups (*P* = 0.5, **Figures 4A, B** and **Supplementary Figures 2B, C**). However, the beta diversity of the REHpp group was significantly lower than that of the REHpn group (*P* = 0.006, R^2^ = 0.053, **Figure 4C**). The PCoA plots revealed separation of the samples in the REHpp and REHpn groups (**Figure 4D**).

### Comparison of Oral Bacterial Abundance Between the REHpp and REHpn Groups

**Figure 5:**
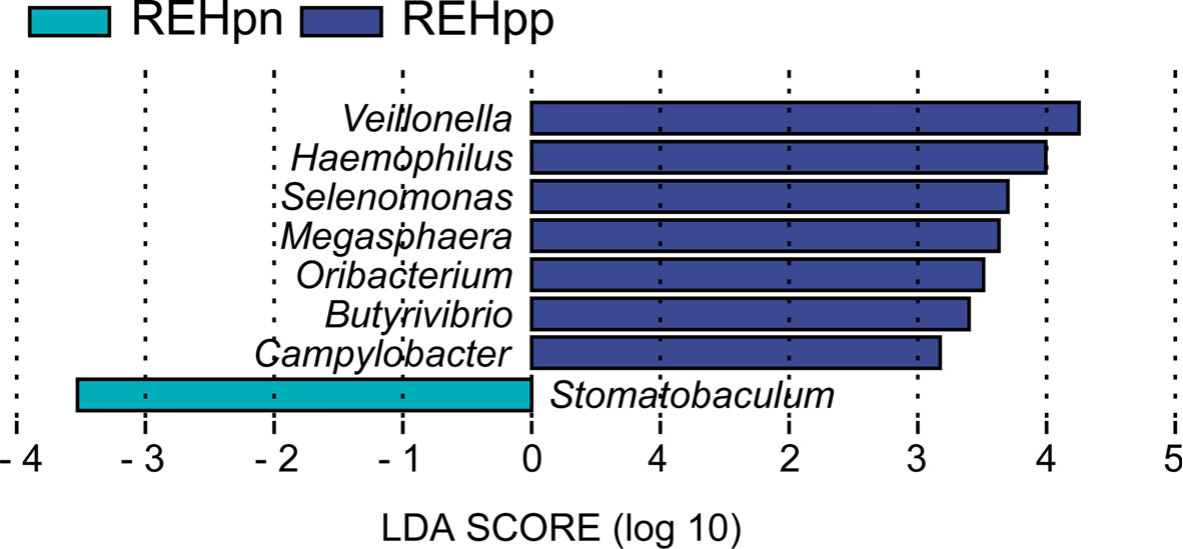
LEfSe analysis shows that the relative abundance rates of *Veillonella*, *Haemophilus*, *Selenomonas*, *Megasphaera*, *Oribacterium*, *Butyrivibrio*, and *Campylobacter* were higher in the REHpp group, while the abundance of *Stomatobaculum* was higher in the REHpn group.

No significant differences in bacteria phyla were observed between the REHpp and REHpn groups (**Supplementary Figure 2D**). At the genus level, the relative abundance rates of *Veillonella* and *Selenomonas* were higher in the REHpp group (*P* < 0.05, **Figures 4E, F**). We performed LEfSe analysis to further confirm the differentially abundant taxa in the REHpn and REHpp groups. At the genus level, the biomarker in the REHpn group was *Stomatobaculum*, while those in the REHpp group were *Veillonella*, *Haemophilus*, *Selenomonas*, *Megasphaera*, *Oribacterium*, *Butyrivibrio*, and *Campylobacter* (**Figure 5**).

### Comparison of the Oral Microbial Network Between the REHpp and REHpn Groups

**Figure 6:**
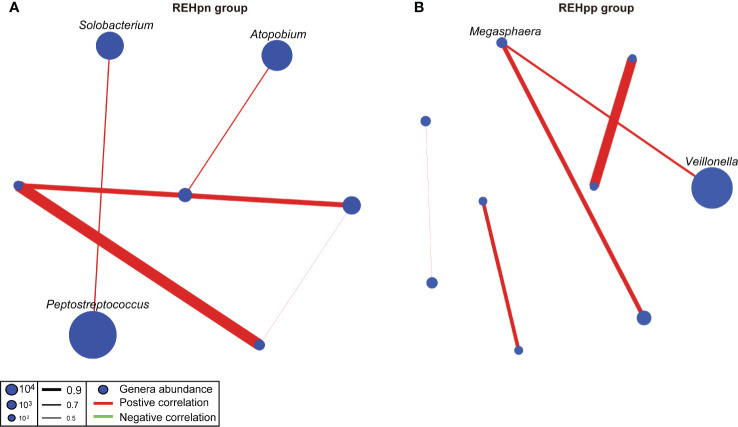
Co-occurrence networks of the REHpn and REHpp groups **(A, B)**. Each node in the network represents a bacterial genus. The node size represents the mean relative abundance of a genus in the oral microbiota. Red line: positive correlation; green line: negative correlation. Relative thickness of the lines represents the degree of correlation (greater thickness of the edges means a stronger correlation).

We compared the oral microbial network of the REHpn and REHpp groups to explore the effect of *Hp* infection on the oral microbial network of RE patients. The microbial network structure of the REHpp group (9 nodes, 5 sides) was similar to the REHpn group (7 nodes, 5 sides). *Megasphaera* was positively correlated with *Veillonella* in the microbial network of the REHpp group. *Solobacterium* was positively correlated with *Peptostreptococcus* in the microbial network of the REHpn group (**Figure 6**). Overall, these results indicate that the microbial network of the oral microbiota of RE patients was affected by *Hp* infection.

## Discussion

The oral microbiota plays a fundamental role in maintaining normal physiology (Gao et al., 2018). Disorders of the oral microbiota may be essential for inducing digestive tract diseases, such as RE, chronic gastritis, and indigestion (Coker et al., 2018; Wang et al., 2020). Wang et al. (2020) reported that oral microbiota disorders are closely related to RE. Many studies have shown a negative association between *Hp* infection and the incidence of RE (Lv et al., 2019; Sugimoto et al., 2020). Recent research suggests that *Hp* is negatively correlated with the incidence of esophageal disease (Anderson et al., 2008). Some scholars hold the view that *Hp* is protective against RE (Nie et al., 2014; Neto et al., 2016). In addition, *Hp* eradication treatment may worsen RE, and promote the occurrence and development of esophageal diseases, such as Barrett’s esophagus (Zhou et al., 2020). In this study, we report a comprehensive characterization of the microbial characteristics that distinguished RE patients from healthy people and emphasize the effect of *Hp* infection on the oral microbiota of RE patients. The results of our study show that RE increases oral microbiota disorders and that *Hp* may inhibit these disorders.

### Effect of Reflux Esophagitis on Alpha Diversity of the Oral Microbiota

The alpha diversity of the oral microbiota of RE patients was similar to that of the healthy control group. Following the present results, previous studies have demonstrated no significant difference between alpha diversity of the oral microbiota of patients with upper gastrointestinal diseases and that of healthy people (Wang et al., 2020). No differences in alpha diversity of the esophageal microbiota are observed between healthy people, Barrett’s esophagus patients, and esophageal adenocarcinoma patients (Lopetuso et al., 2020). Snider’s studies also reported that the alpha diversity of oral microbes in patients with Barrett’s esophagus is not significantly different from that of healthy people (Snider et al., 2018). Hence, we hypothesized that RE may not affect the alpha diversity of the human oral microbiota.

According to these data, we infer that a *Hp* infection slightly increases the alpha diversity of the oral microbiota of RE patients. Current research suggests that the alpha diversity of human oral microbiota may not be affected by *Hp* infection (Chua et al., 2019). Gudra’s research revealed no significant difference in the alpha diversity of the oral and gut microbiota between RE patients and healthy control subjects (Gudra et al., 2020). The alpha diversity of the human intestinal microbiota does not seem to be affected by *Hp* eradication treatment (Gudra et al., 2020). Therefore, *Hp* infection may not be a crucial factor affecting the alpha diversity of oral microbial communities. We suggest that *Hp* infection does not affect the alpha diversity of the oral microbiota in RE patients.

Our results show that the alpha diversity of the oral microbiota in RE patients was slightly lower than that of healthy people. We also observed that the alpha diversity of the REHpp group increased slightly compared with the REHpn group. *Hp* infection may be a protective factor for RE patients, which is consistent with an earlier study (Anderson et al., 2008). Taken together, we speculate that RE may lead to a slight decrease in the alpha diversity of the human oral microbiota, but *Hp* infection may inhibit this decrease. *Hp* infection slightly increased the alpha diversity of the oral microbiota of RE patients, which may stabilize the bacterial community structure.

### Effect of Reflux Esophagitis on Beta Diversity of the Oral Microbiota

This study demonstrated that the beta diversity of RE patients was significantly higher than that of healthy people. Previous studies have reported that RE is a key factor affecting the beta diversity of the human oral microbiota (Wang et al., 2020). Zhou’s study clarified that the oral microbiota community structure of patients with Barrett’s esophagitis is significantly different from that of healthy people, which is consistent with our research results (Zhou et al., 2020). Thus, we consider that the beta diversity of human oral microbiota is affected by RE.

The beta diversity of the REHpp group was significantly lower than that of the REHpn group. *Hp* infection may affect oral microbial community structure, as the beta diversity of the oral microbiota of *Hp* infected patients is significantly different from that of healthy people (Chua et al., 2019; Ji et al., 2020). Beta diversity of the intestinal microbiota changes significantly in humans after *Hp* eradication treatment (Gudra et al., 2020). UniFrac distance is significantly different between patients who have not undergone *Hp* eradication and those who have undergone *Hp* eradication treatment (Gudra et al., 2020). *Hp* causes chronic gastritis to deteriorate into gastric atrophy, intestinal metaplasia, and gastric cancer (Gantuya et al., 2020). The community structure of the gastric microbiota in patients with gastric atrophy, intestinal metaplasia, or gastric cancer is significantly different from that of healthy people (Gantuya et al., 2020). Our results show that the beta diversity of the oral microbiota of RE patients was significantly higher than that of healthy people. However, the beta diversity of oral microbiota of the REHpp group was significantly lower than that of the REHpn group due to *Hp* infection. We speculate that this may have been due to the *Hp* infection, which led to a decrease in beta diversity of the oral microbiota of patients with RE.

### Effect of Reflux Esophagitis on the Abundance of Oral Bacterial Phyla

Our results show that the abundance of Bacteroidetes was higher in the RE group, while the abundance of Firmicutes was higher in the C group. The abundance of Bacteroidetes is lower in the oral and esophageal microbiota of healthy people (Gantuya et al., 2020). Previous studies reported that the abundance of Bacteroides is higher, while the abundance of Firmicutes is lower in the oral and esophageal microbiota of RE and Barrett’s esophagus patients (Gantuya et al., 2020; Lopetuso et al., 2020). Zhou et al. (2020) reported that Bacteroidetes may promote the deterioration of RE into esophageal adenocarcinoma.

The abundance of Firmicutes in the esophagus of healthy people is significantly higher than that in RE patients (Liu et al., 2013). According to one recent report, the abundance of Firmicutes decreases with the severity of esophageal disease (Liu et al., 2013). Compared with patients with high-grade dysplasia of Barrett’s esophagus, the abundance of Firmicutes in patients with Barrett’s esophagus increases significantly in those with non-dysplasia and low-grade dysplasia (Park and Lee, 2020). Liu J. et al. (2021) confirmed that the abundance of Firmicutes is negatively correlated with RORγt, but positively correlated with FoxP3. RORγt and FoxP3 are important transcription factors for Th17 and Treg cells, respectively (Liu J. et al., 2021). An increase in the number of Th17 cells and a decrease in Treg cells are also observed in patients with RE (Liu J. et al., 2021).

Yang’s research suggests that the human esophageal microbiota can be divided into type I and type II (Yang et al., 2009). The healthy people’s esophagus is the type I microbiota, mainly Firmicutes and Gram-negative anaerobes. In contrast, the RE patient’s esophagus is a type II microbiome, and Bacteroides and Gram-negative anaerobes account for high (Yang et al., 2009; Lv et al., 2019). Most of the bacteria from Firmicutes belong to Gram-positive bacteria, and bacteria from Bacteroides belong to Gram-negative bacteria. The abundance of Gram-positive bacteria is higher in *Hp*-infected patients, whereas the abundance of Gram-negative bacteria is higher in non-infected-*Hp* patients (Park and Lee, 2020). The current study argues that the human disease state may promote a change in the body’s microbial community from Gram-positive bacteria to Gram-negative bacteria (Yang et al., 2009). Healthy people have a higher abundance of Gram-positive bacteria in the oral cavity (Okereke et al., 2019). RE causes the numbers of Gram-positive and Gram-negative bacteria in the human oral microbiota to decrease and increase, respectively (Deshpande et al., 2018). The DNA and RNA of Gram-negative bacteria and their metabolite lipopolysaccharide stimulate upregulation of TLR4 expression (Deshpande et al., 2018). TLR4 plays a vital role in the pathogenesis of esophageal diseases (Deshpande et al., 2018). Activation of TLR4 triggers the nuclear factor kappa-B pathway, which leads to inflammation. Moreover, the toxins produced by Gram-negative bacteria can damage the stability of the genome and cause DNA damage and inflammation (Deshpande et al., 2018). We speculate that this may due to RE-induced chronic inflammation, resulting in a decrease in the abundance of Firmicutes and an increase in the abundance of Bacteroidetes.

### Effect of Reflux Esophagitis on the Abundance of Oral Bacterial Genera

Our study shows that the biomarkers in the RE group were *Prevotella*, *Veillonella*, *Leptotrichia*, and *Actinomyces*. It has been assumed that the abundance of *Veillonella* is negatively correlated with the degree of exacerbation of esophageal disease (Snider et al., 2018). Compared with non-dysplastic Barrett’s esophagus patients, patients with high-grade dysplasia Barrett’s esophagus have a lower abundance of *Veillonella* in the oral microbiota (Lopetuso et al., 2020). The abundance of *Prevotella*, *Veillonella*, and *Leptotrichia* in the esophagus of esophageal adenocarcinoma patients increases significantly (Lopetuso et al., 2020). There is some evidence to suggest that *Prevotella*, *Veillonella*, and *Leptotrichia* can interact to induce cancer (Lopetuso et al., 2020). *Leptotrichia* is an opportunistic pathogen associated with many cancers, such as colon cancer, stomach cancer, and pancreatic tumors (Fan et al., 2018; Kim et al., 2018). *Leptotrichia* stimulates immune response and indirectly promotes esophageal tumors (Lopetuso et al., 2020). Bronswijk et al. (2019) suggested that *Actinomyces* is a key genus that induces RE.

Our research found that the biomarkers of the C group were *Lautropia*, *Gemella*, *Rothia*, and *Streptococcus*. *Lautropia* is a key bacterial genus in the oral microbiota that plays an anti-inflammatory effect, and its reduced abundance may lead to the proliferation of other pro-inflammatory bacteria (Vincent et al., 2013; Desai et al., 2016). The abundance of *Lautropia* in the oral cavity of healthy people increases significantly compared to patients with head and neck squamous cell carcinoma (Guerrero-Preston et al., 2016). The abundance of *Lautropia*, *Gemella*, *Rothia*, and *Streptococcus* in the oral microbiota of healthy people is higher compared with patients with RE (Wang et al., 2020). A high abundance of Gram-negative bacteria may promote biofilm formation (Blackett et al., 2013). A high-abundance of Gram-negative bacteria produce lipopolysaccharides and activate the natural immune response, which subsequently stimulates the expression of nuclear factor kappa-B to promote the release of interleukin-1β, -6, -8, and tumor necrosis factor-α (Yang et al., 2012; Kåhrström et al., 2016). Lipopolysaccharides produced by microorganisms enter the blood circulation through the digestive tract mucosa, thereby inducing local and systemic inflammatory reactions, particularly in patients with digestive tract diseases (Zhao et al., 2019).

Our study showed that the biomarker of the oral microbiota in the REHpn group was *Stomatobaculum*; and those in the REHpp group were *Veillonella*, *Haemophilus*, *Selenomonas*, *Megasphaera*, *Oribacterium*, *Butyrivibrio*, and *Campylobacter*. *Hp* infection reduces gastric acid secretion, thereby increasing the abundance of Gram-negative bacteria, such as *Veillonella* and *Haemophilus* (Miyata et al., 2019). *Haemophilus* and *Veillonella* produce more lipopolysaccharides than *Hp* (Sharma and Rao, 2012). Lipopolysaccharides activate TLR4 and promote the nuclear translocation of nuclear factor kappa-B, leading to increased IL-8 gene expression (Smith et al., 2003; Yang et al., 2012). The abundance of other bacterial genera in the bacterial community is affected by the presence of *Hp* (Peng et al., 2017). The abundance of *Veillonella* in the human stomach increases after *Hp* infection (Peng et al., 2017). Other studies have reported that the abundance of *Veillonella* decreases significantly after *Hp* eradication (Tanaka et al., 2005). *Haemophilus* has a function similar to that of *Hp* and is a potentially pathogenic bacterium of non-*Hp* infection gastritis (Gantuya et al., 2019). *Haemophilus* is also the dominant bacteria in the gastric microbiota of gastric cancer patients (Gantuya et al., 2019). Importantly, *Haemophilus* causes the accumulation of nitrite, which promotes the production of carcinogens (Gantuya et al., 2019). *Selenomonas* has been detected on the tongue coating of gastric cancer patients and has been considered a potential biomarker (Rodriguez et al., 2020). The abundance of *Oribacterium* in the human oral cavity increases significantly after *Hp* infection (Ji et al., 2020). The relative abundance of *Stomatobaculum* in the oral microbiota of RE patients is significantly higher (Wang et al., 2020). As a result, we speculate that the abundance of bacterial genera in RE patients is affected by *Hp.*

### Influence of *Hp* Infection on the Oral Microbial Network of Reflux Esophagitis Patients

In our study, *Prevotella* was positively correlated with *Veillonella* in the RE group. *Streptococcus* was negatively correlated with *Selenomonas* and *Campylobacter* in the C group. Smoking is a risk factor leading to RE (Wang K. et al., 2019), and the proportion of smokers in the RE group was significantly higher than that in the C group. Smoking increases nitrate intake, and *Veillonella* converts nitrate into carcinogenic nitrosamines and pro-inflammatory nitric oxide (Jia et al., 2021). Vanhatalo’s studies reported that *Prevotella* and *Veillonella* are positively correlated and are essential markers of early inflammation, leading to an increase in the number of lymphocytes and centrioles (Hérivaux et al., 2021; Vanhatalo et al., 2021). Several recent studies have shown that lactic acid and glucose are metabolized into short-chain fatty acids by *Veillonella*, which promotes mucin synthesis (Chen et al., 2020). *Prevotella* is one of the main bacterial genera that decompose mucin (Ilhan et al., 2020). The findings reported here suggest that *Veillonella* and *Prevotella* may be positively correlated through metabolites.

The relationship between *Streptococcus*, *Selenomonas*, and *Campylobacter* may partly be explained by the fact that *Streptococcus* plays a positive role in reducing the production of peroxides, acids, and lipopolysaccharides in the body (Zhou et al., 2020). *Streptococcus salivarius* inhibits the release of inflammatory factors and exerts an anti-inflammatory effect (Di Giacinto et al., 2005; Laws et al., 2021). *Selenomonas* and *Campylobacter* are potential pathogenic bacteria, which promote the release of IL-6, IL-17, and IL-33 and induce the inflammatory response (Di Pilato et al., 2016; Volgenant et al., 2017; Li et al., 2019). *Streptococcus* competitively inhibits the growth of other bacterial genera in the same community through the metabolite H_2_O_2_ (Zhou et al., 2017; Zhou et al., 2020). Therefore, it is likely that connections exist between *Streptococcus*, *Selenomonas*, and *Campylobacter* in the microbial network of the C group.

Another important finding is that *Solobacterium* was positively correlated with *Peptostreptococcus* in the microbial network of the REHpn group. *Megasphaera* was positively correlated with *Veillonella* in the microbial network of the REHpp group. *Solobacterium* degrades cysteine to produce methyl mercaptan using β-galactosidase and protease (Tanabe and Grenier, 2012; Suzuki et al., 2019). Methyl mercaptan is transported and utilized by *Peptostreptococcus* (Tang-Larsen et al., 1995). *Peptostreptococcus* and *Solobacterium* produce various acids, which lower the pH of the environment, induce local inflammation, and induce digestive tract cancer (Senneby et al., 2017). Current studies suggest that the high abundance of *Peptostreptococcus* may increase the risk of esophageal squamous cell carcinoma (Sung et al., 2020). Thus, *Solobacterium* and *Peptostreptococcus* are positively correlated in the microbial network of the REHpn group.

The lactic acid content and the abundance of lactic acid-producing bacteria are both higher in the esophagus of esophageal cancer patients (Lertpiriyapong et al., 2014; Vinasco et al., 2019; Zhou et al., 2020). Disorders of lactate metabolism can aggravate esophagitis to become esophageal cancer (Lin et al., 2020). Several reports have shown that the abundances of *Megasphaera* and *Veillonella* increase in the oral and stomach microbiota of healthy people after *Hp* infection (Castaño-Rodríguez et al., 2017; Miyata et al., 2019; Wu et al., 2020). Excess acetic acid is converted into acetate and propionate by *Megasphaera* and *Veillonella via* the acrylate pathway and the methylmalonyl-CoA pathway, respectively (Canfora et al., 2019; Scheiman et al., 2019). A strong interaction exists between *Hp* and *Veillonella*, and this may be related to gastric lesions or cancer (Guo et al., 2020). We speculate that the abundances of *Megasphaera* and *Veillonella* in the REHpp group were positively correlated, which may be affected by the *Hp* infection.

## Conclusion

Taken together, our research reveals a trend that RE leads to an increase in the beta diversity of the human oral microbiota, an increase in the abundance of Gram-negative bacteria, and a decrease in the abundance of Gram-positive bacteria. However, *Hp* infection inhibits the change in diversity, and the oral microbiota tends to be normal. However, our study had some limitations. We did not compare the oral microbes before and after *Hp* infection in patients with RE, which may only reflect part of the picture. Future research should focus on the use of transcriptomic, proteomic, metabolomic, and other multi-omics approaches to explore the relationship between the oral microbiota, reflux esophagitis, and *Hp*. It is extremely important to explore the role of the oral microbiota in the occurrence and development of RE.

## Data Availability Statement

The datasets presented in this study can be found in online repositories. The names of the repository/repositories and accession number(s) can be found below: https://ngdc.cncb.ac.cn/gsa, accession number: CRA (CRA004395).

## Ethics Statement

The studies involving human participants were reviewed and approved by Ethics Committee of Xizang Minzu University. The patients/participants provided their written informed consent to participate in this study.

## Author Contributions

LK: attributed to the study design. TL, FL, ZZ, LL, WD, SB, and LM: performed the sample collection and experimental work. TL: finished the date analyses and drafted the manuscript. All authors contributed to the article and approved the submitted version.

## Funding

This research was supported by National Natural Science Foundation of China (31660307), Science and Technology Department Project of Tibet Autonomous Region (No. XZ201801-GB-03).

## Conflict of Interest

The authors declare that the research was conducted in the absence of any commercial or financial relationships that could be construed as a potential conflict of interest.

## Publisher’s Note

All claims expressed in this article are solely those of the authors and do not necessarily represent those of their affiliated organizations, or those of the publisher, the editors and the reviewers. Any product that may be evaluated in this article, or claim that may be made by its manufacturer, is not guaranteed or endorsed by the publisher.
